# The Dual Role of Uric Acid: Pathological Implications Across Chronic Diseases and Current Approaches to Hyperuricemia Management

**DOI:** 10.15190/d.2026.10

**Published:** 2026-06-30

**Authors:** Aiza Zehra, Ayeeza Asghar, Sarah Ali, Muhammad Umair Khalid, Syed Imran Ali Shah

**Affiliations:** ^1^CMH Lahore Medical University, Lahore, Pakistan; ^2^Department of Biochemistry, Azra Naheed Medical Colege, Faculty of Medical Sciences, Superior University, Pakistan

**Keywords:** Uric acid, hyperuricemia, chronic diseases, cardiovascular disease, autoimmune disorders, therapeutic interventions, oxidative stress, chronic kidney disease, CDK, inflammation.

## Abstract

Uric acid is the end product of purine metabolism and plays a dichotomous role in the human body. On one hand, it exerts antioxidant and neuroprotective effects; on the other hand, chronic hyperuricemia has been strongly associated with diseases beyond gout, affecting the cardiovascular, renal, metabolic, autoimmune, and central nervous systems (CNS). Excess uric acid promotes oxidative stress, endothelial damage, neurodegeneration, inflammasome activation, and impairs energy metabolism. It exacerbates autoimmune diseases, such as Systemic Lupus Erythematosus (SLE) and antiphospholipid syndrome (APS), by increasing inflammatory and oxidative damage, leading to greater end-organ damage. The British Society for Rheumatology, European League Against Rheumatism, American College of Rheumatology, and National Institute for Health and Care Excellence (NICE) have all established a “treat-to-target” approach for hyperuricemia with serum urate levels below 6 mg/dL and below 5 mg/dL in severe cases. Allopurinol and Febuxostat, xanthine oxidase inhibitors, are used as first-line pharmacological therapies for the treatment of hyperuricemia, whereas uricosurics and Interleukin-1 (IL-1) inhibitors are preferred in cases of refractory hyperuricemia. Lifestyle modifications, such as the Dietary Approaches to Stop Hypertension (DASH) diet, weight reduction, and smoking cessation, are also recommended for the long-term management of the disease. Sodium-Glucose Cotransporter 2 (SGLT2) inhibitors, selective urate transport inhibitors, and plant-derived anti-inflammatory compounds have emerged as new treatments with promising responses. This review synthesizes the current literature on the multifaceted role of uric acid and emphasizes its systemic implications in chronic diseases. It also outlines the already established management options and new innovative therapies for managing this disease. Understanding this dichotomous role is essential for adopting a precise management approach that balances the protective and pathological effects of uric acid.

## SUMMARY

*1. Introduction*

*2. Pathological Impact*

*3. Uric Acid and Chronic Diseases*

*4. Therapeutic Intervention*

- *4.1 Pharmacological Management (Allopurinol, Febuxostat, Uricosurics)*

- *4.2 Lifestyle and Dietary Modifications*

*5. Future Perspectives and Research Directions*

*6. Conclusion*

## 1. Introduction

Uric acid is the final product of purine breakdown in humans and has been linked to a paradoxical role in different physiological and pathological conditions. While it functions as a potent antioxidant in plasma, excessive accumulation has been implicated in numerous long-term health problems and chronic illnesses. While it is established that elevated uric acid levels have been notorious as one of the principal causes for gout and kidney stones, this paper further discusses how uric acid may also play an important role in the development of conditions like hypertension, cardiovascular disease, chronic kidney disease, autoimmune conditions, and its effects on neurological and executive function. The underlying mechanisms by which it exerts its effects are important to highlight so that therapeutic interventions can be better targeted, more effective, and tailored to the specific pathways involved in disease progression. Uric acid can cross the blood-brain barrier and accumulate in the cerebrospinal fluid, impairing neuronal function and causing cognitive decline^1^. minor elevations in levels have exerted complex effects such as structural brain changes and disruptions in cognitive abilities, such as memory, concentration, and comprehension^2^. In the cardiovascular system, hyperuricemia promotes endothelial dysfunction, atherosclerosis, and myocardial remodeling through mechanisms including oxidative stress, inflammasome activation, and disrupted energy metabolism, discussed in detail below. Through these mechanisms, hyperuricemia is invariably associated with increased risk of myocardial infarction, atrial fibrillation, and ischemia-reperfusion injury^3-5^. Moreover, hyperuricemia has also been observed in connection with metabolic dysfunction, correlating with insulin resistance, obesity, and dyslipidemia^6^. Hyperuricemia has also been implicated in the exacerbation of autoimmune conditions, notably systemic lupus erythematosus (SLE), by amplifying oxidative and inflammatory cascades that contribute to disease progression^7^. All these findings and others, as discussed below, point to the systemic impact of hyperuricemia, beyond its classical associations. Understanding its complex role in various disease mechanisms is crucial for the development of effective treatment strategies.

Organizations such as the British Society for Rheumatology (BSR), the European League Against Rheumatism (EULAR), the American College of Rheumatology (ACR), and the National Institute for Health and Care Excellence (NICE) have developed evidence-based guidelines and recommendations to guide the clinical management of hyperuricemia and its associated symptoms. Treatment goals focus on reducing serum urate to below the saturation threshold for crystal formation, with most authorities advocating a target of <6 mg/dL, and even lower (<5 mg/dL) in patients with severe disease manifestations^8^. Guidelines further emphasize a "treat-to-target" approach involving urate-lowering therapy (ULT) in patients with recurrent attacks, renal dysfunction, comorbidities, or evidence of joint damage^8^. First-line pharmacologic interventions include xanthine oxidase inhibitors such as allopurinol and febuxostat, while uricosuric agents and anti-inflammatory therapies provide additional options depending on patient-specific factors^9^. Beyond pharmacological management, dietary and lifestyle modifications play a crucial role in managing hyperuricemia. While low-purine diets have shown limited success, dietary patterns like the DASH and Mediterranean diets, which address underlying metabolic dysfunction, offer a more impactful approach^10^. Weight reduction, regular physical activity, and smoking cessation are also recommended due to their positive effects on serum urate levels^11^.

Recent advancements in research have broadened treatment possibilities with the introduction of novel agents that act on urate transporters (URAT1), sodium glucose co-transporter 2 (SGLT2), and interleukin signaling pathways, as well as natural anti-inflammatory compounds. These recent advancements reflect a promising role in the systemic management of hyperuricemia, offering new possibilities for more effective, targeted, and individualized treatment approaches^12^.

## 2. Pathological impact

Uric acid, a byproduct of purine breakdown, serves as both a physiological antioxidant and a pathological contributor to disease progression. Excessive accumulation of uric acid, termed hyperuricemia (serum uric acid concentration >7.0 mg/dl)^13^ implicated in a wide range of disorders. These include kidney disease, neurological disease, cardiovascular complications, as well as autoimmune conditions, revealing a complex and often contradictory role in human health^14^. Dysregulation of serum uric acid levels by transporters such as urate transporter 1 (URAT1), glucose transporter 9 (GLUT9), and ATP-binding cassette subfamily G member 2 (ABCG2) results in hyperuricemia. The most common acute presentation of hyperuricemia is gout, the formation of monosodium urate crystal deposits in joints and other body tissues. Understanding the dual nature of uric acid is essential to evaluating its pathological impact and therapeutic potential^15^.

## 3. Uric acid and chronic diseases

**Figure 1:**
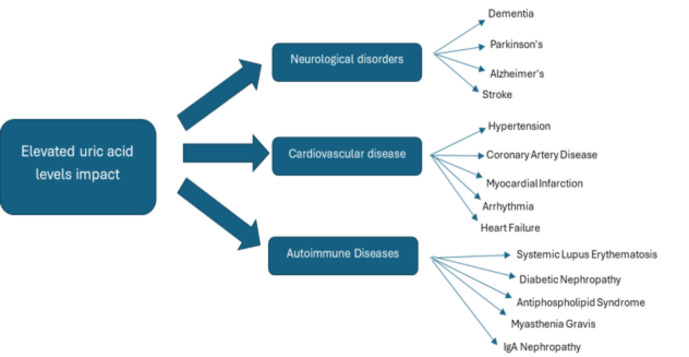
Multisystemic Pathological Impacts of Elevated Uric Acid This directional flow illustration maps the transition of uric acid from a passive biomarker to an active pathophysiological driver across several organ systems

Elevated uric acid levels are increasingly associated with the development and progression of various chronic diseases. From cardiovascular and neurological disorders to kidney disease and autoimmune conditions, uric acid appears to play a complex and often detrimental role, acting as both a biomarker and a potential contributor to disease mechanisms^16^. However, it is important to note that many of these associations are derived from observational studies, and the distinction between correlation and causation remains an active area of investigation. To better understand the role of uric acid, **Figure 1** lists the potential systems and diseases that can be involved.

Hyperuricemia may facilitate the passage of uric acid through the blood-brain barrier from the plasma into the cerebrospinal fluid (CSF), where preclinical evidence suggests it can promote neuroinflammation and neuronal impairment, potentially contributing to cognitive decline^17^. This is particularly important in the context of aging, where even minor fluctuations in uric acid levels may lead to anatomical changes in the brain as well as direct alterations in neural functioning, impairing active memory, concentration, understanding ability, and higher-order cognitive skills^18^.

There is some incongruity between elevated uric acid levels and the severity of central nervous system damage, which shows that other factors may also be involved in the underlying pathology, beyond what is traditionally understood about uric acid’s role. This is corroborated by the fact that certain subtypes of dementia, such as vascular dementia occurring as a complication of underlying stroke, are more prone to hyperuricemia damage due to the damage caused to cerebral blood vessels by elevated uric acid levels^19^, whereas other types of dementia, including Parkinson disease-associated dementia or Alzheimer’s disease, showed no significant variation in levels between cases and controls across multiple studies^2^. Lower serum uric acid levels are correlated with a negative disease course in Parkinson’s disease. Hypouricemia has been associated with a worse clinical trajectory, characterized by decline in motor function, cognitive ability, and overall quality of life over time^20^.

The implications of high uric acid levels in heart disease (**Table 1**) may warrant proactive measures for its management to prevent myocyte damage and lower the risk of cardiovascular disease. **Table 1** outlines the various effects of high uric acid levels on the heart, along with its underlying mechanisms and resulting outcomes^21^.

**Table 1:** The effects of high uric acid levels on the heart

Effects	Results	Mechanism
Direct effects on the myocardium	● Endothelial cell damage^22^	● Decreases troponin affinity for calcium within cardiac myocytes
	● Promotes atherosclerosis	
	● Alterations in myocardial structure	
	● Induction of exudation and fibrosis^23^	
	● Cell shortening^24^	
Inflammasome activation	● Cell swelling and subsequent lysis	● Increased TLR6 levels associated with NLRP3 inflammasome activation^25^
	● Activated macrophages secrete regulatory factors, inducing arrhythmias in surrounding myocytes	● Production of ROS^26^
		● Caspase 1 maturation with subsequent activation of IL-18 and IL-1β and N terminal domain of Gasdermin D (GSDMD)^27^
Disruption of Myocardial Energy Metabolism	● Cardiac hypertrophy	● Insulin resistance in myocytes by insulin receptor substrate 1 phosphorylation, inhibiting Akt phosphorylation and GLUT4 translocation^28^
	● Accumulation of cytoplasmic lipids^29^	● AMPK-ULK1 pathway stimulates autophagy^30^
		● Reduced carnitine palmitoyl transferase 1B activity affects fatty acid oxidation^29^
Diminished antioxidative potential	● Exacerbation of oxidative stress in the myocardium	● Increased levels of malondialdehyde (MDA) and decreased superoxide dismutase (SOD) and catalase (CAT)
		● Downregulation of nuclear factor erythroid 2-related factor 2 (Nrf2) which regulates antioxidant stress^3^
Myocardial Ischemia-Reperfusion Injury	● Hypoxia/	● Reperfusion generates oxygen radicals, activates NLRP3 inflammasome and the ROS/TRPM2/Ca2+ pathway^4^
	● Reperfusion in cardiac cells by the production of reactive oxygen species and cellular proptosis^31^	

The Italian URRAH (Uric Acid Right for Heart Health) study, a multicenter observational cohort (n = 23,467), proposed a prognostic cut-off of >5.7 mg/dL for predicting fatal myocardial infarction (HR 1.38, 95% CI 1.10–1.76), with the association significant in women but not men^32^.

The advancement of coronary artery disease can also be attributed in part to uric acid levels in patients with both comorbidities as elevated uric acid may accelerate plaque disruption, calcification and the formation of hemorrhagic thrombi^33^.

An observational study reported that for each 1 mg/dL rise in uric acid levels, the risk of atrial fibrillation was associated with a 35% increase in women compared with 15% in men^5^. In the ARIC cohort (n = 15,382), per-SD HRs were 1.25 (95% CI 1.08–1.43) in women versus 1.05 (95% CI 0.94–1.18) in men, with a significant interaction (p < 0.01).

Piepoli et al. (2020) analyzed the MECKI database (n = 4,577 patients with heart failure with reduced ejection fraction [HFrEF]) and found serum uric acid was associated with increased total and cardiovascular mortality (HR 1.120 and 1.128, respectively; p < 0.0001). Critically, this association was significant only in New York Heart Association (NYHA) class I–II (HR 1.17, p < 0.0001) and not in class III–IV (HR 1.03, p = NS)^34^.

Damage to heart muscle in this context has been found to correlate with elevated levels of circulatory biochemical markers, including highly sensitive troponin I and N-terminal pro-brain natriuretic peptide (NT-proBNP), further supporting an association between uric acid and deterioration of heart muscle function^35^.

A study conducted in Bangladesh that divided participants based on their blood pressure levels showed that xanthine oxidase levels were significantly higher in hypertensive patients, encompassing even patients on anti-hypertensive medication, than in patients with normal blood pressure levels^36^. through its association with hypertension, hyperuricemia has also been proposed as a potential risk factor for stroke, as suggested by the REGARDS study conducted in 2020, where hyperuricemia was associated with ischemic stroke (HR 1.40, 95% CI 1.10–1.78) after adjustment for demographic variables and blood pressure^37,38^. Alternatively, multiple studies have failed to find an association between uric acid levels and vascular complications or mortality in patients with ischemic stroke^39^.

A cross-sectional study conducted in Japan among adolescents revealed that hyperuricemia was associated with features suggestive of metabolic syndrome, such as increased body mass index, glycosylated hemoglobin levels, non-alcoholic steatohepatitis, and increased low-density lipoprotein (LDL) levels suggestive of hypercholesterolemia^40^.

Xanthine oxidoreductase (XOR), an enzyme crucial for the formation of uric acid from purines, is significantly associated with insulin resistance, which stimulates its activity, providing a plausible biological explanation for why uric acid levels may be higher in patients with diabetes mellitus^41^.

High uric acid levels have been implicated in the progression of several autoimmune diseases, potentially worsening their prognosis by activating inflammatory and oxidative pathways, ultimately targeting end organs involved in the primary disease or amplifying an already toxic internal environment and further disturbing the body’s equilibrium.

**Figure 2:**
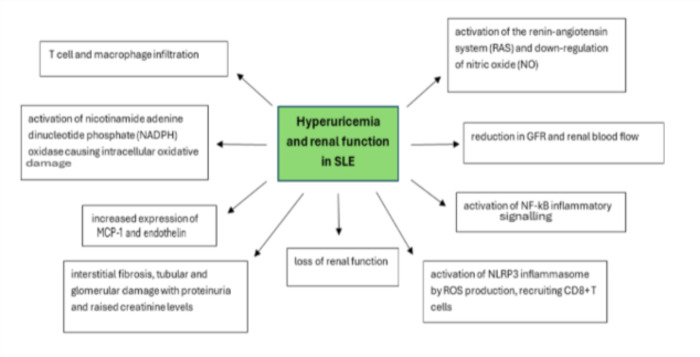
Mechanistic Pathways of Hyperuricemia-Induced Renal Function in SLE This schematic illuminates the synergistic pathways through which hyperuricemia aggravates renal pathology within the inflammatory baseline of SLE, linking the convergence of distinct molecular cascades to functional renal decline.

For example, systemic lupus erythematosus, a well-known autoimmune disease, has been observed to be complicated by hyperuricemia, as explained by the following diagram (**Figure 2**)^7^.

Nephropathy is also aggravated in diabetic patients with hyperuricemia, with levels of uric acid >420 umol/l for men and >360 umol/l for women complicating diabetic nephropathy by further reducing glomerular filtration rate (GFR), causing proteinuria and progressive renal impairment^42^.

A recent cross-sectional study compared uric acid levels and its effects on patients with another autoimmune disease known as antiphospholipid syndrome (APS). It showed that the patients afflicted with APS who had higher serum uric acid levels also had greater occurrences of arterial hypertension, myocardial infarction, thrombotic microvascular disease and disordered lipid metabolism. Despite the small sample size of this study, the selection of patients with APS and no other coexisting conditions reduces confounding factors significantly and makes the complications mentioned above strongly attributable to high uric acid levels^43^.

Lu et al. (2020) cohort studied 208 IgAN patients divided by uric acid levels and found the hyperuricemia group had significantly worse renal outcomes (Kaplan-Meier, p = 0.0147), higher tubular atrophy/interstitial fibrosis scores, higher vascular injury scores, and greater glomerular sclerosis percentage (all p < 0.01)^44^.

A meta-analysis discussing the role of uric acid in myasthenia gravis interestingly found that patients of myasthenia had lower levels of uric acid in their blood compared to healthy individuals, and more severe symptoms were found in patients with lower uric acid levels. However, the dichotomy of uric acid in myasthenia gravis lies in its dual role, while lower levels are linked to greater disease severity, its complex metabolism and potential risks at higher concentrations make it unsuitable as a direct therapeutic target^45^.

Hyperuricemia has also been shown to complicate rheumatoid arthritis (RA)-associated interstitial lung disease (ILD), with higher levels in the serum and bronchoalveolar fluid of RAILD patients, specifically in the setting of usual interstitial pneumonia (UIP)^46^. A larger multicenter study (n = 829) confirmed elevated UA as an independent risk factor for ILD in RA (OR 1.68, 95% CI 1.23–2.28) and a prognostic indicator for mortality (HR 1.80, 95% CI 1.03–3.17)^47^.

The relationship between hyperuricemia and kidney disease is complex and bidirectional, as elevated uric acid levels can both result from impaired renal function and contribute to further kidney injury through multiple pathogenic pathways. Among the renal structures affected by hyperuricemia, the tubular compartment is particularly vulnerable. Renal tubular injury may result from both urate crystal deposition and the direct effects of soluble uric acid. Monosodium urate (MSU) crystals can form within the tubular lumen when urinary uric acid concentrations exceed their solubility threshold and once deposited, these crystals may trigger inflammatory responses and tissue injury. Studies suggest that MSU crystals can activate the NLR family pyrin domain-containing 3 (NLRP3) inflammasome, a multiprotein complex that promotes maturation of pro-inflammatory cytokines such as IL-1β and IL-18. This contributes to inflammasome activation, interstitial inflammation and fibrosis, supporting a pathogenic role for local crystal formation in kidney injury. Soluble uric acid itself can also directly contribute to renal damage by stimulating inflammatory signaling and cytokine production, including IL-1β. In proximal tubular epithelial cells, activation of the NLRP3 inflammasome occurs through TLR4-dependent pathways and may be amplified by oxidative stress, mitochondrial dysfunction, reactive oxygen species generation, lysosomal damage, and endoplasmic reticulum stress. Collectively, these findings indicate that both crystalline and soluble forms of uric acid promote renal tubulointerstitial injury through interconnected inflammatory and oxidative mechanisms, highlighting their potential contribution to the progression of chronic kidney disease (CKD) and the complex relationship between CKD and hyperuricemia^48^.

Endothelial dysfunction is a key pathway through which hyperuricemia contributes to renal injury. Elevated uric acid levels reduce nitric oxide bioavailability by suppressing endothelial nitric oxide synthase activity while simultaneously increasing reactive oxygen species production, leading to oxidative stress^49^ acid also promotes vascular inflammation through activation of hypoxia-inducible factor-1α (HIF-1α), enhancing endothelial permeability and leukocyte recruitment. Together, these effects induce vascular remodeling and impair renal autoregulation, resulting in abnormal glomerular hemodynamics that may accelerate kidney injury and CKD progression^50^. Lastly, hyperuricemia can alter glomerular hemodynamics by affecting the afferent arteriole and impairing endothelial function. This may lead to either glomerular hyperfiltration or hypoperfusion. The hyperfiltration pattern is associated with proteinuria, activation of the renin–angiotensin system, and progressive glomerular injury, whereas the hypoperfusion pattern is characterized by reduced glomerular blood flow and ischemic injury with minimal proteinuria^51,52^. All these findings suggest that elevated uric acid can contribute to CKD progression through distinct hemodynamic mechanisms.

Although uric acid is traditionally viewed as a pathogenic metabolite implicated in gout, cardiovascular disease, chronic kidney disease and metabolic disorders, emerging evidence suggests that its biological role is more complex. Uric acid exhibits a dual nature, functioning not only as a mediator of disease in increased concentrations but also as an important physiological molecule with potential protective effects. A recognized beneficial role of uric acidat normal physiological levels is its inhibition of CD38, an enzyme that breaks down nicotinamide adenine dinucleotide (NAD⁺), a molecule essential for energy production, cellular repair and immune function. By reducing CD38 activity, uric acid helps preserve NAD⁺ levels and prevent excessive inflammation. In contrast, when uric acid levels exceed the normal levels and form monosodium urate (MSU) crystals, these crystals can increase CD38 expression in immune cells, leading to greater inflammation. Therefore, while excessive uric acid can contribute to inflammatory conditions such as gout, normal levels of UA may be beneficial by maintaining NAD⁺ availability and helping regulate immune responses^14^.

Uric acid also contributes significantly to the body's antioxidant defense system. Reactive oxygen species (ROS) play an essential role in regulating cellular metabolism, however excessive ROS production can damage cellular structures such as DNA, proteins, and lipids leading to cell dysfunction, cell death, and various diseases. Uric acid is recognized as an important endogenous antioxidant, and studies have demonstrated that it protects cells from oxidative damage by scavenging harmful free radicals, including peroxyl radicals, hydroxyl radicals, and singlet oxygen. It exerts this antioxidant effect by donating electrons and chelating metal ions, thereby limiting free radical reactions. During the process of ROS neutralization, uric acid is oxidized to allantoin and other metabolites, making allantoin a useful biomarker of oxidative stress in humans. These findings highlight the important role of uric acid in protecting against oxidative stress and suggest its potential contribution to the prevention of aging, cancer, and other oxidative stress-related diseases^14^.

The neuroprotective potential of uric acid has been well recognized and is primarily attributed to its ability to counter oxidative stress. Under normal physiological conditions, uric acid crosses the blood brain barrier only minimally and is produced in low amounts within the brain. However, during neurological disorders, disruption of the blood brain barrier may allow circulating uric acid to enter the central nervous system (CNS), where it can exert protective effects by scavenging reactive oxygen species. Clinical studies have reported an association between reduced uric acid levels and an increased risk of neurological diseases, while accumulating evidence suggests that uric acid may help protect against disorders such as Multiple Sclerosis (MS), Alzheimer's disease and Parkinson's disease. In multiple sclerosis, a disease characterized by demyelination in the CNS, oxidative stress is thought to play a significant role in disease progression, and experimental studies indicate that uric acid administration may help reduce disease severity through its antioxidant actions. Similarly, its precursors such as inosine have demonstrated beneficial outcomes, and clinical trials suggest that inosine supplementation may improve symptoms and disease progression in MS patients^51^. Recent research has also identified increased expression of CD38 in MS, while inhibition of CD38 has been shown to elevate NAD⁺ levels, as discussed previously^52^. This reduces neuroinflammation and slows disease progression. Since uric acid directly inhibits CD38, it may provide neuroprotective benefits in MS by both reducing oxidative stress and preserving the availability of NAD⁺. A study published recently also suggests that monitoring uric acid levels over time may serve as an additional biomarker for understanding treatment response in multiple sclerosis. More effective MS treatments were associated with a greater long-term increase in serum uric acid levels, suggesting that uric acid may be a useful marker of disease control and oxidative stress^53^.

Several pre-clinical study findings also support a potential neuroprotective role of uric acid in Parkinson’s disease. Beyond its antioxidant activity, uric acid may help slow disease progression by modulating the Nrf2-ARE signaling pathway which regulates cellular antioxidant defenses, influencing the Akt/GSK3β signaling pathway involved in neuronal survival and function and promoting mitochondrial remodeling and maintenance, which may protect neurons from degeneration^54^. Several studies have shown an inverse relationship between uric acid levels and Parkinsons Disease risk, with higher uric acid levels being associated with a lower risk of developing the disease. In contrast, diets that elevate uric acid levels and conditions such as gout have been associated with a reduced risk of Parkinson’s disease^55^.

*Influence of Sex, Age, Ethnicity, and Comorbidity Burden on Uric Acid and Disease Outcomes*

Serum uric acid levels are consistently higher in males than females during reproductive years, primarily due to the uricosuric effect of estrogen, which enhances urate excretion by upregulating intestinal ABCG2 expression through the PI3K/Akt signaling pathway^56^. However, this sex gap narrows substantially after menopause. NHANES 2007–2018 data (n = 34,144) demonstrated that while overall hyperuricemia prevalence was higher in males (21.1%) than females (17.1%), females surpassed males in both prevalence and absolute numbers after age 50–59^57^. A longitudinal study of 8,169 Japanese women confirmed that serum urate rose sharply during perimenopause (mean increase 0.41 mg/dL by post menopause), with approximately 18% of overweight or obese women at menopause developing hyperuricemia^58^.

Beyond prevalence, women with hyperuricemia appear to face disproportionately higher cardiovascular risk at equivalent uric acid levels. A German population-based study (DEGS1, n = 6,918) found that the prevalence of cardiovascular and renal diseases increased with higher uric acid levels to a much greater extent in women than in men^59^. Sex-specific risk factor profiles also differ in the NHANES analysis, alcohol consumption was positively associated with hyperuricemia in males but not females, while diabetes was protective in males (OR = 0.72) but a risk factor in females (OR = 1.22).

Gout prevalence increases with age, and older patients frequently present with multiple comorbidities that modify disease outcomes. Among patients with three or more cardiac-renal-metabolic conditions, hyperuricemia and poorly controlled gout showed significant positive associations with all-cause mortality, whereas these associations were not observed in patients with gout who had achieved normal serum urate levels^60^. Older patients are also more susceptible to allopurinol hypersensitivity syndrome, particularly in the setting of CKD and diuretic use, necessitating more cautious dose titration^61^.

Significant ethnic disparities exist in hyperuricemia prevalence and genetic susceptibility. In the NHANES analysis, Non-Hispanic Black adults had the highest hyperuricemia prevalence (23.9% in males, 23.4% in females). These disparities are partly explained by population-specific differences in urate transporter gene variants. The ABCG2 Q141K loss-of-function variant (rs2231142) has been associated with a 1.75-fold increase in gout risk across multiple populations, with the minor allele frequency being higher in East Asian populations^62^.

Ethnicity also influences treatment response. In the STOP Gout trial, non-White race was associated with significantly lower odds of achieving serum urate goal with treat-to-target therapy (aOR 0.32–0.47), independent of adherence and baseline serum urate^63^. These findings underscore the need for ethnicity-informed management strategies and further investigation into the biological and social determinants of treatment disparities.

## 4. Therapeutic interventions

Both the British Society for Rheumatology and British Health Professionals in Rheumatology (BSR/BHPR), as well as the European League Against Rheumatism (EULAR), have issued comprehensive guidelines for the management of gout, offering clear benchmarks for clinical practice. Additionally, the National Institute for Health and Care Excellence (NICE) has provided evidence-based recommendations tailored to the UK healthcare setting, ensuring that therapeutic strategies are aligned with both international and national standards. These guidelines form the foundation for evaluating and optimizing current treatment approaches in gout^64^.

One of the primary goals in the treatment of gout is to lower serum uric acid levels beyond the threshold where urate crystals can form and deposit, triggering the pathological process. Since the formation of urate is a reversible process, a serum uric acid level of below 6 mg/dL is the target^65^. Although the 2017 British Society for Rheumatology (BSR) guidelines and the 2022 National Institute for health and Care Excellence (NICE) guidelines differ in their recommended target serum uric acid levels for therapy, both ultimately align in their overarching treatment approach aimed at long-term disease control and reduction of gout flares^66^. Other management strategies have been outlined by the 2021 American College of Rheumatology (ACR) and the 2016 European League Against Rheumatism (EULAR) with similar goals^67^. **Table 2** briefly summarizes the recommendations offered by NICE, ACR, EULAR, and BSR.

**Table 2:** A comparison and summary of NICE, ACR, EULAR, and BSR guidelines to manage hyperuricemia

Strategy	2017 BSR	2022 NICE	2021 ACR	2016 EULAR
Target serum urate level	<360 µmol/L (6 mg/dL); lower target of <300 µmol/L (5 mg/dL) for tophaceous or severe disease	<360 µmol/L (6 mg/dL); lower target of <300 µmol/L (5 mg/dL) conditional on tophi, chronic gouty arthritis, or persistent frequent flares despite <6 mg/dL	<360 µmol/L (6 mg/dL)	<360 µmol/L (6 mg/dL); lower target of <300 µmol/L (5 mg/dL) particularly for severe disease (tophi, chronic symptoms, frequent flares)
Indications to start ULT in gout	Recommends discussing ULT with all patients from the first attack of gout, using a treat-to-target approach	Diagnosis of gout with a treat-to-target approach	Established gout with ≥2 flares/year, tophaceous gout, or radiographic damage due to gout. Conditionally recommended for first flare with CKD ≥ stage 3, SUA >9 mg/dL, or urolithiasis	Recurrent or complicated gout. Recommends discussing ULT close to the time of first diagnosis in patients <40 years, SUA >8 mg/dL, or with comorbidities (renal impairment, hypertension, ischemic heart disease, heart failure)
Asymptomatic hyperuricemia	ULT not recommended for asymptomatic hyperuricemia	ULT not recommended for asymptomatic hyperuricemia	Conditionally recommended against initiating ULT in asymptomatic hyperuricemia, including those with comorbid CKD, CVD, urolithiasis, or hypertension	ULT not recommended for asymptomatic hyperuricemia
Flare frequency threshold	May initiate after first or recurrent flare	Frequent flares	≥2 flares/year	Recurrent flares (≥2/year)
Tophi/extent of damage	Tophaceous gout; chronic gouty arthritis	Chronic gouty arthritis	Tophi observed clinically or through imaging; radiographic damage due to gout	Urate-related joint disease; tophi
Renal involvement	Mentions CKD specifically (stages G3–G5); recommends lower starting dose of allopurinol in CKD	Mentions CKD stage 3–5 (G3–G5) specifically	CKD stage ≥3 (conditional indication for ULT after first flare)	Kidney dysfunction as a whole
Urolithiasis	History of renal stones considered an indication for ULT	Not explicitly mentioned, but falls under individuals who have experienced gout flares that do not fall into the categories mentioned above	History of urolithiasis (conditional indication for ULT after first flare)	Mentions kidney stones as an indication for ULT
Special populations requiring ULT	May initiate after first or recurrent flare; emphasizes early intervention	May initiate after first or recurrent flare	–	Young age (<40 years), SUA >8 mg/dL (480 µmol/L), and/or comorbidities: hypertension, coronary artery disease, heart failure
First-line agent in ULT	Allopurinol (start low, go slow: 100 mg/day if eGFR >60; 50 mg/day if eGFR <60; dose escalation in 50–100 mg increments monthly to target). Febuxostat as alternative if allopurinol not tolerated or contraindicated	Allopurinol OR Febuxostat. Prefer allopurinol in patients with cardiovascular disease (previous MI, stroke, unstable angina)	Allopurinol (preferred first-line, including in CKD ≥ stage 3). Febuxostat as alternative. Probenecid as first-line if prior two not tolerated. Emphasizes HLA-B*5801 testing in Southeast Asian and African American patients	Allopurinol is the first-line ULT for patients with normal kidney function
*HLA-B5801 testing*	Recommends testing prior to starting allopurinol in patients of Southeast Asian or African descent	Not explicitly addressed	Strongly recommends HLA-B5801 testing prior to allopurinol in Southeast Asian and African American patients	Recommends HLA-B*5801 testing prior to allopurinol in at-risk populations
Anti-inflammatory prophylaxis during ULT initiation	Recommends colchicine prophylaxis (up to 6 months) when starting ULT	Recommends anti-inflammatory prophylaxis	Strongly recommends concomitant anti-inflammatory prophylaxis for at least 3–6 months	Recommends prophylaxis with colchicine (first-line) for 6 months
Timing of ULT initiation	Can be started during an acute flare	Can be started during an acute flare	Recommends starting during an acute flare (with anti-inflammatory treatment)	Can be started during an acute flare

### 4.1. Pharmacological Interventions

As mentioned previously, experts across the board and most guidelines recommend several drug classes for the management of hyperuricemia and gout. These include Xanthine Oxidase inhibitors, for example, Allopurinol and Febuxostat, that work to inhibit the enzyme Xanthine Oxidase and thus prevent the formation of uric acid cellularly^68^. Uricosuric agents such as Probenecid, Dotinurad, and Benzbromarone each work to facilitate uric acid excretion from the body via different mechanisms. Some studies suggest that their usage is linked to an increased risk of acute kidney injury^69^ but safer alternatives in terms of dosing, and drug options such as Dotinurad 4 mg, once daily, or Verinurad have also demonstrated lesser adverse effects and better efficacy^70^. Anti-inflammatory therapy includes the use of non-steroidal anti-inflammatory (NSAID) medication, corticosteroids, and colchicine. Studies support the efficacy of NSAIDs and underscore the similar effectiveness of low-dose colchicine as compared to high-dose colchicine. Steroids may be administered orally, via the intra-articular route, or intramuscularly. Although there is a lack of placebo-controlled trials for the use of both oral and intramuscular steroids, multiple randomized controlled trials support their efficacy in the setting of acute gout^71^. Amongst agents that inhibit interleukin 1, a principal mediator in the pathogenesis of gout^72^. Studies have demonstrated the superiority of Canakinumab. It is an anti-interleukin 1 beta monoclonal antibody that reduces pain and inflammation in patients where the use of NSAIDs and colchicine is contraindicated^73^. Alternative options such as Anakinra and Rilonacept were analyzed in a systematic review, which declared the latter as more effective in managing pain and reducing the frequency of acute flares. Anakinra seems to be as effective as other treatments, though this may be influenced by how it works, the small number of studies, and differences in study designs^74^_._

### 4.2. Dietary and Lifestyle Modifications

Before any pharmacological intervention, unless indicated, dietary and lifestyle modifications are essential for the long-term management of gout^75^. While evidence supporting the efficacy of conventional low-purine diets in preventing hyperuricemia remains limited, poor patient adherence due to low palatability further undermines their clinical utility^76,77^. A more pragmatic approach involves targeting modifiable metabolic factors with broader relevance. Notably, the well-documented association between hyperinsulinemia and hyperuricemia suggests that addressing insulin resistance could yield dual benefits^78^. In this context, dietary interventions such as the DASH or Mediterranean diet, which are both sustainable and metabolically favorable, may serve as effective strategies for managing hyperuricemia in patients with concurrent metabolic dysfunction^79^.

The Mediterranean diet emphasizes daily intake of whole grains, fruits, vegetables, legumes, nuts, and olive oil, which are rich in fiber and antioxidants. Moderate amounts of dairy (especially yogurt and cheese), fish, poultry, and eggs are included regularly, while red meat is limited to small monthly portions. Red wine in moderation (up to 2 glasses/day for men, 1 for women) is preferred for its antioxidant content. The results of three separate studies on the effectiveness of the Mediterranean diet on hyperuricemia and gout yielded promising results, with an overall decrease in serum uric acid levels^80^.

The DASH diet, originally designed to manage hypertension, emphasizes whole grains, fruits, vegetables, low-fat dairy, and plant-based proteins from legumes and nuts instead of animal sources^81^. This diet has also produced favorable outcomes for those suffering from hyperuricemia^10^.

Furthermore, lifestyle modifications such as weight reduction, smoking cessation, and overall adherence to healthy habits have been shown to lower the risk and incidence of hyperuricemia. Weight loss in overweight or obese individuals lowers serum uric acid levels by improving insulin sensitivity and enhancing renal excretion of uric acid. Regular physical activity supports gout management by lowering inflammation, improving joint mobility, and promoting cardiovascular health. Moderate-intensity exercises like walking, swimming, or cycling are preferred, as they help prevent flares without overloading the joints. Smoking contributes to systemic inflammation and elevated cardiovascular risk, both of which can worsen gout symptoms and complicate management. Quitting smoking is strongly recommended, as it reduces flare frequency, enhances treatment effectiveness, and improves overall health outcomes^82^.

## 5. Future Perspectives and Research Directions

Transparency in the role of UA in the development of many pathophysiological conditions has allowed for the development of targeted therapeutic interventions that help modulate UA levels in the body. While xanthine oxidase inhibitors (XOIs) such as allopurinol and febuxostat remain first-line agents for reducing serum urate levels, pharmacotherapy directed at URAT1 inhibitors, SGLT2 inhibitors, immunotherapeutics, and plant-derived compounds represents a rapidly expanding therapeutic landscape^83^.

*URAT1 inhibitors*

**Benzbromarone**, a non-selective URAT1 inhibitor available in many countries but not FDA-approved due to rare hepatotoxicity, has shown cardiovascular benefit in a large Korean cohort (HR 0.82 for composite cardiovascular events vs. allopurinol; HR 0.60 for all-cause mortality^84^.

**Lesinurad**, a selective URAT1/OAT4 inhibitor, was FDA-approved in 2015 for combination use with a XOI. In RCTs, lesinurad 200 mg/day combined with a XOI achieved approximately 1 mg/dL greater serum uric acid reduction than XOI alone^85^. However, serum creatinine more than doubled in 1.8% of patients at the approved dose, and efficacy is impaired at eGFR <30 mL/min. It was subsequently withdrawn from the US market for business reasons. CYP2C9 metabolism necessitates caution with co-prescribed CYP2C9 inhibitors.

**Dotinurad** is a novel selective urate reabsorption inhibitor (SURI) approved in Japan. It demonstrated the highest URAT1 selectivity among available uricosurics (IC₅₀ 0.0372 µM vs. 0.190 µM for benzbromarone and 30.0 µM for lesinurad), with minimal effects on secretion transporters ABCG2 and OAT1/3^86^. In a phase 3 trial in Chinese gout patients, dotinurad 4 mg/day was superior to febuxostat 40 mg/day in achieving serum urate ≤6.0 mg/dL at week 24 (73.6% vs. 38.1%; p<0.0001)^87^. Notably, the renal safety event incidence was only 0.5%, substantially lower than lesinurad (11.2%) and verinurad (12.0%). Exploratory data from the DIANA study suggest potential vascular benefits (improved arterial stiffness and oxidative stress markers), though confirmation in larger trials is needed.

**Epaminurad** is currently in phase 2b development. It demonstrated dose-dependent efficacy in a multicenter RCT of 169 gout patients: 88.89% (9 mg), 71.79% (6 mg), and 54.05% (3 mg) achieved serum urate <6 mg/dL at week 4, compared with 0% for placebo (all p<0.0001). The 9 mg response rate was comparable to the febuxostat 80 mg reference arm (84.21%), with no significant differences in adverse events versus placebo^88^.

**Darbinurad** is another URAT1 inhibitor in clinical development, though published trial data remain limited. Other earlier-stage agents include **verinurad** (phase 2; potent efficacy but 12.0% renal safety event rate)^89,90^, **URC-102** (phase I; dose-dependent uric acid reduction in healthy volunteers)^91^, and **HP501** (phase I/IIa; up to 50% serum uric acid reduction with synergistic effects when combined with febuxostat)^92^.

A model-based meta-analysis of 49 studies (10,591 participants) comparing drug classes found comparable urate-lowering efficacy between URAT1 inhibitors (37.5% reduction) and XOIs (35.4%), with URAT1 inhibitors showing lower adverse event rates (51.8% vs. 55.8%) and lower dropout rates (8% vs. 17%). However, eGFR declined by 2.5% after one year with URAT1 inhibitors versus a 0.7% increase with XOIs, highlighting the importance of renal monitoring^93^.

*SGLT2 Inhibitors*

Moreover, SGLT2 inhibitors have recently shown proficiency in lowering UA levels, thereby attenuating the progression to renovascular disease^94^. SGLT2 inhibitors lower uric acid by approximately 0.6–1.5 mg/dL through transactivation of GLUT9-mediated urate efflux, URAT1 downregulation, and potential ABCG2 upregulation^95^. A meta-analysis of 11 RCTs confirmed significant serum uric acid reduction (MD −0.56 mg/dL, 95% CI −0.66 to −0.46, p<0.00001)^93^.

*Uric acid, hypertension, and atrial fibrillation*

When building on the hypothesis that the uricase gene mutation, which occurred in the hominids, is coupled with modern era high salt dietary habits, which can lead to earlier onset of hypertension, studies are being conducted to elucidate the effect of UA lowering agents on hypertension before the emergence of microvascular injury^96,97^. A meta-analysis of RCTs demonstrated a favorable effect of urate-lowering treatment on systolic blood pressure (mean difference −2.55 mmHg, 95% CI −4.06 to −1.05), though large-scale outcome trials are needed^98^. Adding to this, the strong correlation between atrial fibrillation (AF) and HUA has paved the way for studies on the role of urate-lowering therapy in decreasing the incidence of AF^99^. A drug-target Mendelian randomization study found genetic evidence supporting URAT1 inhibition for reducing AF risk (OR 0.91, 95% Bayesian credible interval 0.85–0.97), though mediation analyses suggest the benefit may be partly indirect through reductions in hypertension (56% mediation) and heart failure (48%)^100^.

*Plant-derived compounds and immunotherapeutic approaches*

More recently, natural plant-based compounds are being studied to exert anti-inflammatory effects on mice with gouty arthritis (GA), as flavonoids and carotenoids are known for their role in hampering inflammation^101^. Moreover, usage of immunotherapeutics like inhibitors of IL-6 receptor signaling can diminish MSU-induced IL-1β production, disrupting the progression of autoinflammatory conditions like gout^102^. Ongoing studies also talk about targeting ROS, more specifically ROS-autophagy, ROS-NET, ROS-ferroptosis, with ROS being the common denominator for inflammation associated with GA, but their therapeutic significance is yet to be determined^103,104^.

*Major challenges and future directions*

Several challenges may limit clinical translation of emerging therapies:

**Renal safety:** Uricosuric agents increase urinary uric acid excretion, raising nephrolithiasis risk (pooled incidence 3.35%; RR 1.63 vs. non-uricosuric treatments)^104^. Newer selective agents (e.g., dotinurad, 0.5% renal events) show markedly improved profiles compared with older agents (lesinurad, 11.2%)^96^. Combination with XOIs can reduce renal uric acid excretion by 48%, mitigating this risk.

**Drug-drug interactions:** CYP2C9 metabolism (lesinurad) and non-selective transporter inhibition (benzbromarone) pose interaction risks in the polypharmacy settings common among gout patients. Newer selective agents appear to have minimal transporter effects.

**Patient selection:** Uricosuric efficacy depends on adequate renal function (most not recommended at eGFR <30 mL/min). Phenotyping patients as urate underexcretors versus overproducers could guide therapy selection but is not routine practice.

**Long-term outcomes and cost-effectiveness:** Long-term data on gout flare reduction, tophus resolution, cardiovascular events, and renal preservation remain limited for most newer agents. Urate-lowering therapy has not yet demonstrated consistent cardiovascular benefit in RCTs (OR 0.67, 95% CI 0.44–1.03 for major adverse cardiovascular events [MACE]). Comparative cost-effectiveness analyses will be essential as newer agents enter the market.

Future research should prioritize large-scale RCTs evaluating long-term clinical outcomes of emerging therapies, the development of dual-target XOR/URAT1 inhibitors, integration of SGLT2 inhibitors into gout management algorithms, and translation of preclinical findings on plant-derived and ROS-targeted therapies into human studies.

## 6. Conclusion

This review has demonstrated the dual role of uric acid as both a protective and pathogenic entity across multiple organ systems. At physiological concentrations, uric acid functions as a potent endogenous antioxidant. It scavenges peroxyl radicals, hydroxyl radicals, and singlet oxygen and contributes to immune regulation through inhibition of CD38 and preservation of NAD⁺ availability. However, when levels exceed the physiological threshold, hyperuricemia promotes inflammation, endothelial dysfunction, oxidative stress, and end-organ damage through pathways involving NLRP3 inflammasome activation, nitric oxide depletion, and crystal-mediated tissue injury.

In the cardiovascular system, hyperuricemia has been associated with endothelial damage, atherosclerosis, myocardial remodeling, and arrhythmogenesis through mechanisms including inflammasome activation, disruption of myocardial energy metabolism, and diminished antioxidative potential. In the kidney, both crystalline and soluble forms of uric acid promote tubulointerstitial injury through interconnected inflammatory and oxidative mechanisms, with endothelial dysfunction and altered glomerular hemodynamics contributing to CKD progression. Hyperuricemia has also been implicated in the exacerbation of autoimmune conditions, including systemic lupus erythematosus, antiphospholipid syndrome, IgA nephropathy, and rheumatoid arthritis-associated interstitial lung disease, by amplifying inflammatory and oxidative cascades that target end organs. In the metabolic domain, the association between xanthine oxidoreductase activity and insulin resistance provides a plausible biological link between hyperuricemia and metabolic syndrome.

The neurological implications of uric acid are particularly noteworthy for their paradoxical nature. Hyperuricemia facilitates the passage of uric acid through the blood-brain barrier and promote neuroinflammation and cognitive decline, particularly in the context of vascular dementia, but at the same time, lower serum uric acid levels have been consistently associated with worse outcomes in neurodegenerative diseases. In Parkinson's disease, preclinical evidence suggests that uric acid may slow disease progression by modulating the Nrf2-ARE signaling pathway, influencing the Akt/GSK3β pathway involved in neuronal survival, and promoting mitochondrial remodeling. Several epidemiological studies have demonstrated an inverse relationship between uric acid levels and Parkinson's disease risk, with higher levels associated with reduced disease incidence. Similarly, in multiple sclerosis, uric acid may confer neuroprotective benefits through both its antioxidant actions and its inhibition of CD38, thereby preserving NAD⁺ availability and reducing neuroinflammation. Recent longitudinal data further suggest that serum uric acid trajectories may serve as a biomarker for treatment response in MS. This duality, where uric acid is protective in the central nervous system yet harmful in the cardiovascular and renal systems, represents one of the most clinically significant and unresolved aspects of uric acid biology.

The clinical management of hyperuricemia is guided by international consensus, with the BSR, EULAR, ACR, and NICE guidelines. All of these guidelines advocate a treat-to-target approach with serum urate levels below 6 mg/dL and below 5 mg/dL in severe disease. Xanthine oxidase inhibitors remain first-line urate-lowering therapy. Lifestyle modifications including the DASH and Mediterranean diets, weight reduction, and smoking cessation also serve as important adjunctive strategies. The therapeutic options are rapidly expanding with newer selective URAT1 inhibitors such as dotinurad and epaminurad demonstrating promising efficacy and improved renal safety profiles, and SGLT2 inhibitors offering urate-lowering effects alongside established cardioprotective and nephroprotective benefits. Preclinical studies on plant-derived anti-inflammatory compounds, including flavonoids and carotenoids, have shown potential in modulating gout-related inflammation through inhibition of NF-κB activation and NLRP3 inflammasome components. Separately, biologic agents targeting key inflammatory cytokines, such as IL-1 inhibitors (anakinra, canakinumab) and IL-6 receptor antagonists (tocilizumab), represent distinct immunotherapeutic strategies for crystal-induced arthritis refractory to conventional treatment. Additionally, ROS-targeted therapies addressing interconnected pathways of autophagy, NETosis, and ferroptosis are gaining momentum, though their clinical significance remains to be established.

Despite these advances, several important limitations and unresolved questions warrant consideration. First, many of the associations between hyperuricemia and chronic diseases discussed in this review are derived from observational studies, and the distinction between correlation and causation remains an active area of investigation. Establishing definitive causality will require well-designed prospective studies and Mendelian randomization analyses. Second, the optimal serum uric acid threshold that balances antioxidant benefits against pathological risks has not been established, and it remains unclear whether this threshold varies across disease states and patient populations. Third, long-term data on gout flare reduction, tophus resolution, cardiovascular events, and renal preservation remain limited for most newer pharmacological agents, and urate-lowering therapy has not yet demonstrated consistent cardiovascular benefit in randomized controlled trials. Fourth, the conflicting roles of uric acid across different organ systems raise fundamental questions about whether systemic urate-lowering strategies may inadvertently compromise neuroprotective effects while addressing cardiovascular and renal risk.

Future research should prioritize large-scale randomized controlled trials evaluating long-term clinical outcomes of emerging therapies, the development of dual-target XOR/URAT1 inhibitors, integration of SGLT2 inhibitors into gout management algorithms, and translation of preclinical findings on plant-derived and ROS-targeted therapies into human studies. Precision medicine approaches that tailor urate-lowering therapy based on genetic, metabolic, and disease-specific profiles may ultimately be necessary to navigate the complex and often contradictory biological effects of uric acid across the spectrum of chronic disease.

## Declaration of Generative AI and AI-assisted technologies

In the course of preparing this work, the author(s) utilized ChatGPT and Gemini to enhance language clarity, grammar, and conciseness. These tools were employed solely for language enhancement, and not for generating any of the scientific ideas, interpretations, analysis, or conclusions presented in this paper. After using these tools, the author(s) reviewed and edited the paper as needed, and therefore, take full responsibility for the final content.

## Publisher’s note

All claims expressed in this article are solely those of the authors and do not necessarily represent those of their affiliated organizations, or those of the publisher, the editors and the reviewers. Any product that may be evaluated in this article, or claim that may be made by its manufacturer, is not guaranteed or endorsed by the publisher.
